# Effect of dietary advanced glycation end products on inflammation and cardiovascular risks in healthy overweight adults: a randomised crossover trial

**DOI:** 10.1038/s41598-017-04214-6

**Published:** 2017-06-23

**Authors:** Estifanos Baye, Maximilian PJ de Courten, Karen Walker, Sanjeeva Ranasinha, Arul Earnest, Josephine M Forbes, Barbora de Courten

**Affiliations:** 1 Monash Centre for Health Research and Implementation, School of Public Health and Preventive Medicine, Monash University, Melbourne, Australia; 2 Centre for Chronic Disease, College of Health and Biomedicine, Victoria University, Melbourne, Australia; 3 Department of Nutrition and Dietetics, School of Clinical Sciences, Monash University, Melbourne, Australia; 4 Biostatistics Unit, School of Public Health & Preventive Medicine, Monash University, Melbourne, Australia; 5 Mater Clinical School, University of Queensland, Brisbane, Australia; 6 Glycation and Diabetes, Mater Research Institute–University of Queensland, Translational Research Institute, Brisbane, Australia; 7 Diabetes and Vascular Medicine Unit, Monash Health, Melbourne, Australia

## Abstract

Diets high in advanced glycation end products (AGEs) are thought to be detrimental to cardiovascular health. However, there remains uncertainty about the beneficial effect of a low AGE diet on cardiovascular risk factors and inflammatory markers in overweight individuals. We thus performed a randomised, double blind, crossover trial to determine whether consumption of low AGE diets reduce inflammation and cardiovascular risks in overweight and obese otherwise healthy adults. All participants (n = 20) consumed low and high AGE diets alternately for two weeks and separated by a four week washout period. Low AGE diets did not change systolic (p = 0.2) and diastolic blood pressure (p = 0.3), mean arterial pressure (p = 0.8) and pulse pressure (p = 0.2) compared to high AGE diets. Change in total cholesterol (p = 0.3), low-density lipoprotein (p = 0.7), high-density lipoprotein (p = 0.2), and triglycerides (p = 0.4) also did not differ and there was no difference in inflammatory markers: interleukin-6 (p = 0.6), monocyte chemoattractant protein-1 (p = 0.9), tumour necrosis factor α (p = 0.2), C-reactive protein (p = 0.6) and nuclear factor kappa beta (p = 0.2). These findings indicate that consumption of low AGE diets for two weeks did not improve the inflammatory and cardiovascular profiles of overweight and obese adults.

## Introduction

Advanced glycation end products (AGEs) are formed endogenously from non-enzymatic reactions of amino acids with sugars^1^. Consumption of foods high in sugar and/or foods exposed to high temperature cooking methods such as deep-frying, broiling, roasting, baking and grilling can increase the total daily AGE intake by 25% compared to the average adult daily intake^2, 3^. High AGE intake from food accelerates the production of endogenous AGEs and increases the level of circulating AGEs in the blood stream^4, 5^. This intake of high AGE contributes to the progression of type 2 diabetes (T2DM) and cardiovascular diseases (CVD) although the exact mechanisms are not clearly understood^6–11^. AGE formation can be reduced by shortening the cooking time and lowering temperature to reduce food browning, or by addition of acidic (low pH) ingredients and by high humidity or food moisture content^7, 12^. Given the ease of such changes, reduction of AGE intake may be a promising intervention to lower cardiovascular risk^3, 13–15^.

In humans, the impact of low AGE diets on inflammation and cardiovascular risk factors is not clearly understood. In some studies involving healthy obese individuals, low AGE diets reduced inflammatory markers^11, 16, 17^ but did not change plasma lipid levels^17, 18^ or blood pressure^17, 19^. Whereas, other studies showed a reduction in plasma lipid levels after low AGE diets^19–21^ but not of markers of inflammation^18, 22^. In patients with T2DM, low AGE diets improved inflammatory markers^6, 8, 9^, but not blood pressure^9^, and lipid levels^6, 9^. Cai and colleagues however reported a reduction in oxidised low-density lipoprotein after 6 weeks of consumption of low AGE diets (5 fold lower in AGE content) in patients with T2DM^23^. In all these studies, test and control diets were either not matched for energy and macronutrient content or it was not stated whether similar macronutrient content was achieved between the diets, which could potentially influence the results^24^. We have therefore investigated the impact of low and high AGE diets carefully matched for both energy and macronutrient profile. These matched diets were given for two weeks each to healthy obese adults in a randomised cross-over design trial to determine effects on cardiometabolic parameters. We have previously reported the main outcomes of the trial which showed that dietary AGEs improved insulin sensitivity but not insulin secretion in overweight and obese non-diabetic individuals^24^. In this study, we conducted analyses of secondary outcomes of the trial and investigated if dietary AGEs improve blood pressure, plasma lipid profiles and inflammatory markers.

## Results

### Baseline characteristics

As previously described^24^, the mean age, body mass index, waist-to-hip ratio and % body fat of the participants were 34 (10) years, 31.3 (3.8) kg/m^2^, 0.9 (0.1) and 31.1 (6.7), respectively. There was no difference in levels of circulating AGEs and metabolic profiles such as insulin sensitivity and secretion between group 1 and 2 before each test diet^24^. Anthropometric and blood pressure measurements, plasma lipid levels and inflammatory markers were also not different between the two interventions at the beginning of each dietary period (Table 1). The cumulative dietary AGE intakes were significantly lower in the low AGE dietary period compared to the high AGE dietary period (all p < 0.002), as reported previously^24^. The mean consumption of carboxymethyllysine, carboxyethyllysine and methylglyoxal-derived hydroimadazolidine were decreased by 27%, 38%, and 21%, respectively, in the low AGE group compared to the high AGE group.

**Table 1:** Participants’ baseline characteristics at the beginning of each study period (n = 20).

Parameters	Low AGE group	High AGE group	P-value
Weight (kg)	94.2 ± 15.2	94.1 ± 14.3	0.94
Body mass index (kg/m^2^)	31.4 ± 3.8	31.3 ± 3.7	0.85*
% body fat	30.7 ± 6.5	30.6 ± 6.6	0.83
Waist-to-hip ratio	0.9 ± 0.07	0.9 ± 0.07	0.35*
Systolic BP (mm Hg)	123.1 ± 10.8	122.3 ± 12.3	0.66
Diastolic BP (mm Hg)	77.9 ± 8.9	76.7 ± 9	0.38
Mean arterial pressure (mm Hg)	92.9 ± 9.1	91.9 ± 9	0.37
Pulse pressure (mm Hg)	45.1 ± 6.4	45.6 ± 10.1	0.90*
Total cholesterol (mmol/l)	4.8 ± 1	4.6 ± 1	0.06
LDL (mmol/l)	3.1 ± 0.8	3.1 + 0.7	0.61
HDL (mmol/l)	1 ± 0.2	1 ± 0.2	0.35
Triglycerides (mmol/l)	1.5 ± 1	1.3 ± 0.7	0.11*
CRP (mg/l)	2.1 ± 1.9	2.4 ± 2.7	0.69*
TNFα (pg/ml)	2.8 ± 3.7	1.6 ± 0.9	0.18
MCP-1 (pg/ml)	126 ± 99	121 ± 66	0.92*
IL-6 (ng/ml)	1.5 ± 2.2	1.6 ± 2.4	0.64*
NFκβ p65 activity (ng/µg protein)	14 ± 10	12 ± 10	0.53

All values are presented as means ±SDs. Paired t-tests were conducted to determine the p-values. *Data transformation was done. BP, blood pressure; CRP, C-reactive protein; HDL, high-density lipoprotein; IL-6, interleukin -6; LDL, low-density lipoprotein; MAP, mean arterial pressure; MCP-1, monocyte chemoattractant protein-1; NFκβ; Nuclear factor kappa beta; PP, pulse pressure.

### Effect of low AGE diet on inflammatory markers

Inflammatory markers such as tumour necrosis factor α (TNF α), C-reactive protein (CRP), monocyte chemoattractant protein 1 (MCP-1) and interleukin 6 (IL-6) levels and nuclear factor kappa beta (NFκβ) activity in peripheral blood mononuclear cells (PBMCs) did not change after consumption of either low or high AGE diets. Between groups comparison also showed that the changes in these markers were not significantly different between low and high AGE diets (Table 2).

**Table 2:** Effects of low and high AGE diets on markers of inflammation and cardiovascular risk factors (n = 20).

Parameters	Change from baseline in low AGE group		Change from baseline in high AGE group		Difference between the groups	
	Mean ± SD	P-value†	Mean ± SD	P-value†	Mean ± SD	P-value††
Weight (kg)	−0.69 ± 1.34	0.03	−0.16 ± 1.17	0.54	−0.52 ± 1.52	0.17
BMI (kg/m^2^)	−0.22 ± 0.45	0.03	−0.06 ± 0.38	0.49	−0.16 ± 0.5	0.20
% body fat	−0.06 ± 1.2	0.82	−0.08 ± 1.39	0.80	+0.02 ± 1.56	0.37
WHR	+0.004 ± 0.03	0.55	−0.004 ± 0.032	0.54	+0.008 ± 0.031	0.49
Systolic BP (mm Hg)	−1.2 ± 7.7	0.49	−1.3 ± 8.3	0.47	+0.1 ± 11.4	0.24
Diastolic BP (mm Hg)	−0.8 ± 8.4	0.67	−1.9 ± 6.2	0.18	+1.1 ± 11.6	0.33
Pulse Pressure (mm Hg)	−0.4 ± 7.7	0.81	+0.5 ± 9.7	0.80	−0.9 ± 9.2	0.16
MAP (mm Hg)	−0.9 ± 7.3	0.57	−1.7 ± 5.2	0.16	+0.8 ± 10.7	0.80
Total cholesterol (mmol/l)	−0.4 ± 0.5	0.0006	−0.15 ± 0.3	0.04	−0.3 ± 0.6	0.26
LDL (mmol/l)	−0.3 ± 0.3	0.002	−0.01 ± 0.3	0.86	−0.3 ± 0.5	0.73
HDL (mmol/l)	−0.06 ± 0.1	0.03	−0.008 ± 0.08	0.66	−0.05 ± 0.1	0.21
Triglycerides (mmol/l)	−0.1 ± 0.3	0.08	−0.2 ± 0.3	0.01	+0.04 ± 0.5	0.44
IL-6 (ng/ml)	−0.4 ± 1.9	0.71*	−0.2 ± 1.8	0.94*	−0.2 ± 2.9	0.62*
MCP-1 (pg/ml)	−5.4 ± 37.4	0.52	+1.9 ± 44.6	0.84	+7.41 ± 65.8	0.94
TNFα (pg/ml)	−0.4 ± 4.4	0.84*	+1.8 ± 5.5	0.16*	−2.2 ± 7.1	0.15*
CRP (mg/l)	+0.08 ± 0.66	0.57	+0.19 ± 1.55	0.57	+0.1 ± 1.6	0.60
NFκB p65 activity (ng/µg protein)	−3.4 ± 9.9	0.14	−1.3 ± 11.4	0.61	−2.1 ± 16.7	0.18

†Paired t-tests were conducted to determine the p-values within each groups. ††A 2 × 2 ANOVA for cross over study was used to determine the overall difference between the diets. *Data transformation was done. BP, blood pressure; CRP, C-reactive protein; HDL, high-density lipoprotein cholesterol; IL-6, interleukin -6; LDL, low-density lipoprotein; MAP, mean arterial pressure; MCP-1, monocyte chemoattractant protein-1; NFκβ; nuclear factor kappa B; SD, standard deviation.

### Effect of low AGE diets on cardiovascular parameters

Anthropometric measures such as weight and body mass index were significantly reduced only in the low AGE group compared to high AGE group. Percentage body fat and waist-to-hip ratio did not change after consumption of either diet. Between groups comparison have shown that none of these anthropometric measures were different between the diets (Table 2). Systolic and diastolic blood pressure and mean arterial pressure decreased after consumption of both diets whereas pulse pressure was reduced only after intake of low AGE diets. Between groups analyses (after controlling for period and sequence effect) did not show any significant change in these measurements between the diets (Table 2).

The total plasma cholesterol levels decreased in both groups, however, the change between the two diets was not significant. Both plasma low-density lipoprotein (LDL) and high-density lipoprotein (HDL) cholesterol levels decreased with low AGE diet but not changed with high AGE diet whereas the overall difference between the diets was not significant (Table 2). Plasma triglyceride levels decreased with high AGE diet but there was no difference between low and high AGE diets (Table 2).

### Correlation analyses between inflammation markers and anthropometric measures and cardiovascular risk factors

The correlation analyses indicated that the change in diastolic blood pressure and MCP-1 is positively correlated with % body fat (r = 0.43, p = 0.05) and waist-to-hip ratio (r = 0.46, p = 0.03), respectively. Change in systolic blood pressure is also positively correlated with NFκβ (r = 0.47, p = 0.04) whereas plasma lipid levels negatively correlate with CRP (total cholesterol: r = −0.53, p = 0.01; HDL: r = −0.59, p = 0.006; LDL: r = −0.56, p = 0.008) and IL-6 (total cholesterol: r = −0.51, p = 0.03; HDL: r = −0.58, p = 0.01; LDL: r = −0.54, p = 0.01). After adjusting either for change in weight, body mass index, % body fat or waist-to-hip ratio, neither systolic blood pressure nor lipid parameters correlated with inflammatory markers. Other inflammation parameters did not show any correlation with cardiovascular parameters (all p > 0.1). Circulating and urinary AGE levels also did not correlate with any cardiovascular parameters (all p > 0.09).

## Discussion

The present study aimed to measure the effect of consuming low AGE diets for 2 weeks on markers of inflammation and cardiovascular risk factors in healthy obese and overweight individuals. No significant difference was found in changes in blood pressure, plasma lipid levels (total cholesterol, LDL, HDL, triglycerides) or inflammatory markers (CRP, TNFα, MCP-1, IL-6, NFκβ activity) between low and high AGE diets. Our findings on blood pressure measurements were consistent with other studies conducted in healthy overweight individuals^17, 19^, prediabetes^21^, obese people with metabolic syndrome^25, 26^, and patients with T2DM^9^.

Regarding plasma lipid profile, we did not find a significant difference between low and high AGE diets. Some studies conducted in healthy individuals^17, 18^, obese individuals with metabolic syndrome^25, 26^, and patients with T2DM^6, 9^ have also reported that consumption of low AGE diets did not change plasma lipid levels compared to high AGE diets. In contrast, two other studies that involved young healthy obese volunteers^20^ and prediabetic individuals^21^ noted a reduction in plasma lipid levels after a low AGE diet. In the first study, the difference between the 2 diets could have been due to the higher intake of dietary fat and carbohydrate on the high AGE diets^20^. The patients in the second trial in the low AGE arm of the study had relatively lower intima-media thickness compared to those who were in the high AGE diet^21^. This may exaggerated the effect of high AGE diets on lipid profile. In addition, consumption of low AGE diets combined with aerobic exercise program for 12 weeks showed a decrease in plasma lipid levels in overweight and obese men^19^. This effect however could have been due to the synergistic effect of physical activity with low AGE diets as it has been widely known for its beneficial effect on metabolic changes^27^. Therefore, these studies^19–21^ were not able to differentiate between the true effect of low and high AGE diet as they were confounded by other differences in diet, exercise or disease state of the patients.

In this study, intake of low AGE diets for 2 weeks did not change inflammatory markers. This finding was consistent with some studies conducted in healthy overweight and obese individuals^18, 22^. Other studies conducted in healthy individuals showed an improvement in inflammatory markers after intake of low AGE diets^11, 16, 17^. However, in these studies, the diets were not matched for macronutrient content, which might have influenced the findings. Studies in obese people with metabolic syndrome^26^, prediabetes^21^ and patients with T2DM^8, 9^ showed an improvement in inflammatory markers after intake of low AGE diets compared to high AGE diets. Therefore, it could be suggested that intake of low AGE diets was beneficial for those who had higher baseline values in inflammatory markers such as patients with metabolic syndrome or T2DM.

Compared to the previous studies, our study has several strengths that include standardised diets in energy and macronutrient content were provided, study personnel including the investigators were unaware of the dietary allocation, and baseline characteristics such as anthropometric, circulating AGEs and metabolic parameters were not different at baseline during each dietary period. We have also used a crossover design to control for possible confounding factors in addition to the rigorous randomisation process. The small sample size and relatively short duration could be considered as main limitations of this study as one can argue that the change in physiologic processes after intake of low AGE diets may require longer duration.

In conclusion, we have shown that consumption of low AGE diets for 2 weeks did not have beneficial effects in reducing markers of inflammation and cardiovascular risk factors in healthy overweight and obese adults. Long-term well designed trials with larger sample sizes are needed to confirm our findings.

## Methods

### Study participants

Twenty overweight and obese but otherwise healthy and normoglycemic adults, aged 18–50 years completed the study^24^, according to a protocol as previously published elsewhere^28^. Participants did not have diabetes as indicated by a 75 g oral glucose tolerance test (OGTT) (WHO 1999 criteria). All were non-smokers and had no clinical and laboratory signs of infection and none took supplements/medications during the study period. The participants were recruited from the general community through advertisements between January 2006 and December 2010. Ethical approval was obtained from the Alfred Hospital Ethics Committee, Melbourne, Australia and complied with the Declaration of Helsinki. All participants provided written informed consent prior to participation.

### Study design

This study employed a two-period randomised cross-over double-blind design. All participants underwent both diets; one low in AGE content and one high in AGE content (typical of a modern Western diet) and diets were matched in energy and macronutrient content. Participants commenced the study after a two-week run-in on their habitual diet but with limitation of their alcohol, fast food and coffee intake. Test diets were consumed for two weeks separated by a four-week washout period (habitual diet). The primary outcomes of this study that focused on insulin resistance/sensitivity have been published^24^.

### Randomisation and masking

Randomisation occurred for 7 blocks of 4 participants stratified by gender and diet order. Participants were masked to the allocation of diet type and to how the diet might affect glucose metabolism. Clinical and laboratory investigators were also masked to diet allocation.

### Study Procedures

All participants underwent medical and laboratory screening including a 75 g oral glucose tolerance test (OGTT). Prior to metabolic testing, participants were asked to abstain from strenuous exercise and caffeine for 3 days.

### Dietary Intervention

Prior to starting the ‘run-in’ to the first diet, participants kept a 3-day diet record of their habitual diet (2 weekdays, 1 weekend day) based on household measures. Nutrient content was analysed with SERVE (SERVE Nutrition Systems, St Ives, NSW), based on Australian food composition tables. With the use of Australian food composition data from SERVE as well as data from the United States on the N (ε)-carboxymethyllysine (CML – the most common AGEs used in clinical studies as an indicator of dietary AGE intake) content of common foods, a menu of carefully matched alternative food choices (of low AGE vs high AGE content) individualised to suit the preferences and habitual diet of each participant was designed. These alternative food choices were matched for macronutrient content and total energy but greatly differed in calculated AGE content, and were provided for each meal of the day, including snacks and beverages. Food choices on the high AGE diet had higher dietary AGE content, while the isoenergetic low AGE diet, matched for macronutrient content, had reduced AGE content through altered cooking techniques^4^ and use of differently processed foods. All food for the two test diets was provided weekly to the participants as ready-to-eat items or as packed food portions, to assist with dietary compliance. For food that required cooking, detailed instructions for storage and heating (method, temperature, and duration) were provided. Participants were asked to eat to appetite throughout both dietary periods to maintain constant body weight. They were required to keep a detailed dietary record indicating cooking method and number of portions eaten for each food item supplied, unconsumed foods or additional foods eaten. Later foods incorporated in these diets were also chemically analysed for their AGE content. The dietician made telephone contact twice a week to provide support and resolve difficulties as well as to ensure dietary compliance. To analyse the dietary AGE content, food items were obtained from local supermarkets and prepared according to the instructions provided to the participants in the study.

### Cardiovascular measures

Systolic and diastolic blood pressure were measured using an automated oscillometric measurement system (Omron) after a 30 minute rest. Pulse pressure was calculated by subtracting the diastolic blood pressure from the systolic blood pressure. Mean arterial pressure was computed as diastolic blood pressure + pulse pressure/3^29^. Plasma lipid levels such as total cholesterol, LDL, HDL and triglycerides were measured using a standard commercial enzymatic assay (Beckman Coulter LX20PRO Analyser and Synchron) and Systems Lipid and Multi Calibrators (Beckman Coulter Diagnostics).

### Measurement of inflammatory markers and AGEs

Markers of inflammation (TNFα, MCP-1 and IL-6 levels) were analysed using a commercial automated chemiluminescent enzyme immunoassay and immulite analyser (Diagnostic Products Corporation). Whereas, highly sensitive near infrared particle immunoassay rate methodology was used to measure plasma CRP levels. NFκβ activity in PBMCs was detected and quantified using TransAM NF- κβ DNA-binding activity assay (Active Motif, Carlsbad, CA, USA). Measurement of urinary and serum AGEs were quantified with the use of ultraperformance liquid chromatography–tandem mass spectrometry as previously described^30^.

### Statistical analysis

Change from baseline values was calculated for each parameter in both dietary periods. Paired t-tests were used to compare the participants’ characteristics at the beginning of each dietary period and to determine the change in parameters after each study diet. Repeated measures ANOVA was employed to determine the change difference between the two diets after adjusting for carryover, sequence and period effects. The observed carryover effect in this study was very minimal (29.8%) and thus the data in both study periods were combined. Results are presented using means and standard deviations unless and otherwise stated. Appropriate data transformation was undertaken in the event of skewed data to approximate a normal distribution. Statistical analyses were performed using Stata 14 software. P-value of ≤ 0.05 is used to describe statistical significant difference.

### Data Availability

The datasets generated during and/or analysed during the current study are available from the corresponding author on reasonable request.
